# Shenmai Injection enhances short-term outcomes in ischemic stroke patients after thrombolysis via AMPKα1

**DOI:** 10.3389/fphar.2025.1552493

**Published:** 2025-05-01

**Authors:** Jing Wu, Zhonghao Li, Xiaoke Dong, Jinmin Liu, Le Wang

**Affiliations:** ^1^ Department of Rehibition, Dongfang Hospital Beijing University of Chinese Medicine, Beijing, China; ^2^ Department of Neurosurgery, Dongfang Hospital Beijing University of Chinese Medicine, Beijing, China; ^3^ Department of Neurology, Beijing Daxing District Hospital of Integrated Chinese and Western Medicine, Beijing, China; ^4^ Department of Neurology, Dongzhimen Hospital Beijing University of Chinese Medicine, Beijing, China; ^5^ Department of Neurology, Dongfang Hospital Beijing University of Chinese Medicine, Beijing, China

**Keywords:** Acute ischemic stroke, shenmai injection, AMPK, modified rankin scale, randomized controlled trial

## Abstract

**Background:**

Shenmai Injection (SMI), a traditional Chinese medicine with nourishing properties, has been explored for its therapeutic effects in ischemic stroke (IS). This study aimed to evaluate the protective effects of SMI in patients with IS who received intravenous thrombolysis and to elucidate its potential molecular mechanisms through laboratory investigations.

**Methods:**

Patients with IS were randomized to receive either SMI or a placebo for 10 days within 12 h post-intravenous thrombolysis. Clinical efficacy and safety were assessed. An IS cell model was induced using H2O2, followed by treatment with SMI to explore its therapeutic effects and underlying mechanisms.

**Results:**

The modified Rankin Scale (mRS) score at 30 days was significantly lower in the SMI group (n = 35) compared to the placebo group (n = 35), indicating improved functional outcomes. No significant difference was observed in NIHSS scores between the groups. Adverse events and biochemical indices showed no significant differences, confirming the safety of SMI. In the H2O2-induced cell model, SMI enhanced cell viability, reduced apoptosis, and decreased the levels of malondialdehyde (MDA) and reactive oxygen species (ROS). It also improved ATP content and mitochondrial membrane potential. Mechanistic studies revealed that these protective effects were partially mediated through the AMPKα1.

**Conclusion:**

SMI significantly improves short-term outcomes in IS patients treated with rt-PA thrombolysis. Its protective effects are likely mediated through the AMPKα1, highlighting its potential as an adjunctive therapy for IS.

## 1 Introduction

Ischemic stroke (IS) is an important contributor to mortality and long-term disability, significantly impacting global health (Tang et al., 2021); (Powers, 2020). While timely restoration of blood supply via thrombolytic therapy is considered the main therapy for IS (Cai et al., 2021), the subsequent reperfusion process following ischemic attack may further aggravate brain injury, a phenomenon known as ischemia/reperfusion injury (Dai et al., 2020); (Pérez-Mato et al., 2019). It is increasingly recognized that a variety of pathophysiologic processes, including apoptosis, oxidative stress, and mitochondrial dysfunction play a key role in the occurrence and development of this injury (Jiao et al., 2020; Hu et al., 2020). Therefore, treatments based on the above mechanisms are considered as a promising strategy for alleviating the outcome of IS.

Shenmai injection (SMI), a traditional Chinese medicine injection approved by the China Food and Drug Administration in 1995, has gained recognition for its organ-protective properties (Hu et al., 2020; Yu et al., 2019; Wang et al., 2019). Comprising aqueous extracts from red ginseng (Hong Shen) and Ophiopogonis Radix (Mai Dong), SMI has demonstrated potential in improving clinical outcomes for IS patients. Previous studies have shown that SMI can reduce neurological deficits, lower rates of deterioration, and enhance quality of life (Liu et al., 2017; Xu et al., 2019). However, limitations in study design, including inadequate control groups and unclear treatment protocols, have hindered the comprehensive understanding of SMI’s clinical benefits and mechanisms of action.

Emerging evidence suggests that the AMPKα1 plays a pivotal role in energy metabolism and cellular protection, making it a promising target in mitigating oxidative stress and mitochondrial dysfunction in IS (Chen et al., 2018; Li et al., 2020). Despite its potential, the mechanism through which SMI exerts its protective effects in IS remains inadequately understood.

Taken together, this study aimed to address these gaps by employing a rigorous randomized controlled clinical trial to evaluate the clinical efficacy and safety of SMI in IS patients receiving intravenous thrombolysis. Additionally, the study explored the role of SMI in modulating oxidative stress, mitochondrial dysfunction, and apoptosis, with a focus on the AMPKα1. By integrating clinical and mechanistic insights, this research seeks to provide robust evidence for the therapeutic potential of SMI in IS and contribute to the development of novel treatment strategies.

## 2 Methods

### 2.1 High performance liquid chromatography (HPLC) analysis of SMI

SMI is a commercial injection prepared from the extracts of Panax ginseng C.A.Mey [Araliaceae; Ginseng radix et rhizome rubra] and Ophiopogon japonicus (Thunb.) Ker Gawl [Asparagaceae, Ophiopogonis radix] (Zhang et al., 2023). It was purchased from Chiatai Qingchunbao Pharmaceutical Co., Ltd (Hangzhou, China). The active ingredients present in the SMI include ginsenoside, amino acids, and small molecule organic acids (Li et al., 2022). The quality of the SMI was found to be stable and uniform, exhibiting minimal between-batch variations (Yang et al., 2023).

The composition of SMI was analyzed using HPLC. The HPLC conditions were as follows: Column: Waters Symmetry Shield™ RP18 (4.6 mm × 250 mm, 5.0 µm). Column temperature: 30°C. Mobile phase: Acetonitrile (A) and water (B) with gradient elution (0–30 min: 0% A→10% A, 30–40 min: 10% A→23% A, 40–50 min: 23% A, 50–85 min: 23% A→60% A, 85–95 min: 60% A→100% A). Flow rate: 1 mL/min. Detection wavelength: 203 nm. Injection volume: 10 μL. The fingerprint of SMI is available in Supplementary Figure S1.

### 2.2 Study design

This clinical investigation was designed as a randomized, double-blind, placebo-controlled, parallel-group trial. The study protocol was reviewed and approved by the Ethics Committee of Dongfang Hospital (approval number: JDF-IRB-2020032802). The trial was conducted in accordance with the Declaration of Helsinki and adhered to Good Clinical Practice (GCP) guidelines. The trial was registered with the Chinese Clinical Trial Registry (registration number: ChiCTR2000040106).

### 2.3 Participants

Between January 2021 and January 2022, 72 patients diagnosed with IS who received intravenous rt-PA thrombolysis within 12 h of symptom onset at Dongfang Hospital Beijing University of Traditional Chinese Medicine, were enrolled in this study. Informed consent was obtained from all participants or their legal guardians. Patients with significant comorbidities affecting other organ systems or those with known allergies to any component of the study medication (SMI) were excluded from the study.

### 2.4 Interventions and comparisons

Each participant received an intravenous infusion of either 100 mL of SMI or 0.9% saline solution within 12 h following the rt-PA administration, with treatment continuing for 10 consecutive days. Both treatment groups received standardized supportive care, which included blood pressure management, intracranial pressure reduction, maintenance of fluid and electrolyte balance, and other symptomatic treatments, as per established clinical guidelines.

SMI (batch number: 6901990180090, 100 mL/bottle), which contains 10 g each of Red Ginseng and Ophiopogonis Radix, was produced by Zhengda Qingchunbao Pharmaceutical Co., Ltd. SMI is listed in the Chinese Pharmacopoeia and approved by the China Food and Drug Administration (CFDA) (National Pharmaceutical Approval number: Z33020018). The 0.9% saline solution (batch number: H13023202, 100 mL/bottle) was produced by Shijiazhuang No. 4 Pharmaceutical Co., Ltd.

### 2.5 Randomization, allocation concealment and blinding

Randomization was performed using SPSS version 22.0 software, which generated random numbers that were then equally allocated into two groups. The allocations were transcribed onto distribution cards, which were placed in opaque, sealed envelopes to ensure concealment. Upon enrollment of eligible participants, the envelopes were sequentially opened in accordance with the order of participant inclusion. The randomization procedure was conducted by individuals who were not involved in the study itself.

To maintain blinding, the SMI and 0.9% saline bottles were wrapped in black opaque bags, and an opaque infusion device was used, ensuring that both patients and researchers were unaware of the treatment allocation. During the study, the personnel responsible for data collection and outcome assessment were also blinded to the group assignments, minimizing the potential for bias.

### 2.6 Outcome measurements

The primary outcome measure was the modified Rankin Scale (mRS) score at 30 days, which was used to evaluate short-term prognosis in patients.

The secondary outcome measure was the National Institutes of Health Stroke Scale (NIHSS) score on day 10, which assesses neurological impairment.

Additional outcome measures included biochemical indices assessed on day 10, as well as the monitoring of adverse events and any abnormal changes in biochemical parameters during treatment, to evaluate the safety profile of SMI.

### 2.7 Cell cultures and treatment

The neuron-like rat pheochromocytoma cell line (PC12) was cultured in Dulbecco’s Modified Eagle Medium (DMEM) supplemented with 10% fetal bovine serum (FBS) in a humidified incubator set to 5% CO_2_ and 37°C. Oxidative stress was induced by adding hydrogen peroxide (H_2_O_2_) to glucose-free medium, followed by 24 h of incubation. Subsequently, SMI was added to the culture medium, and cells were incubated for an additional 24 h.

PC12 cells were seeded in 6-well plates and transfected with either si-AMPKα1 (Santa Cruz Biotechnology, Inc., United States). or a negative control small interfering RNA (si-NC) using Lipofectamine 2000 (ThermoFisher Scientific, United States) in accordance with the manufacturer’s protocol. The transfection process was carried out over 24 h, after which cells were harvested for subsequent experiments.

### 2.8 CCK-8 assay

To determine the optimal concentrations of H_2_O_2_ and SMI for further experiments, cell viability was assessed using the Cell Counting Kit-8 (CCK-8) assay (CK04, Dojindo, Japan). In brief, 10 µL of CCK-8 reagent was added to each well, followed by incubation for 2 h. Absorbance was measured at 450 nm using an automated microplate reader (ELX-800, BioTek Instruments, United States).

### 2.9 TUNEL staining

PC12 cells were seeded in 6-well culture plates. After treatment, cells were fixed in 4% paraformaldehyde for 30 min. Subsequently, cells were permeabilized with 0.3% Triton X-100 in phosphate-buffered saline (PBS) for 5 min and incubated with TUNEL detection solution at 37°C for 1 h (C1089, Beyotime, China). Fluorescent images were captured using a BX71 fluorescence microscope (Olympus, Japan).

### 2.10 Oxidative stress assessment

Intracellular reactive oxygen species (ROS) levels were measured using the fluorescent probe 2′,7′-dichlorofluorescein diacetate (S0033S, Beyotiem, China). Fluorescence images were captured using a fluorescence microscope, as described previously. The levels of malondialdehyde (MDA), and superoxide dismutase (SOD) in PC12 cells were quantified using specific commercial assay kits, following the manufacturers’ protocols (Cat. No.: S0131S, S0101S, respectively, Beyotime, China). Absorbance values were measured with a microplate reader, and the results were normalized to total cellular protein content, determined using a bicinchoninic acid (BCA) assay (P1511, Solarbio, China).

### 2.11 Mitochondrial function assessment

Mitochondrial membrane potential was evaluated using JC-1 dye, in conjunction with commercial assay kits, as per the manufacturers’ instructions (C2006, Beyotime, China). Fluorescence images were captured using a fluorescence microscope, as described previously. Adenosine triphosphate (ATP) levels were measured using specific commercial kits (S0026, Beyotime, China), with absorbance values recorded by a microplate reader. ATP levels were normalized to total cellular protein content, as determined by the BCA assay.

### 2.12 Immunofluorescence staining

PC12 cells were fixed with 4% paraformaldehyde for 30 min and permeabilized with 0.3% Triton X-100. Following permeabilization, the cells were blocked with 5% bovine serum albumin blocking buffer and incubated with AMPK alpha 1 primary antibody (5,831, Cell Signaling Technology, United States) overnight at 4°C. Subsequently, cells were incubated with a CoraLite488-conjugated goat anti-rabbit IgG secondary antibody (SA00013-2, proteintech, China) at 37°C for 1 h, followed by staining with DAPI for 5 min. Fluorescent images were acquired using a fluorescence microscope.

### 2.13 Statistical analysis

Statistical analyses were performed using SPSS version 22.0. Categorical variables were described using frequencies and percentages, while continuous variables were summarized using means and standard deviations or medians and quartiles, depending on data distribution. Comparisons between groups for continuous variables were conducted using the t-test or Wilcoxon rank-sum test, and categorical variables were analyzed using the chi-square test or Fisher’s exact test as appropriate. Logistic regression analysis was employed to examine associations between risk factors and outcome events. A significance level of 0.05 was applied to all statistical tests.

## 3 Results

### 3.1 Patient recruitment and baseline characteristics

**FIGURE 1:**
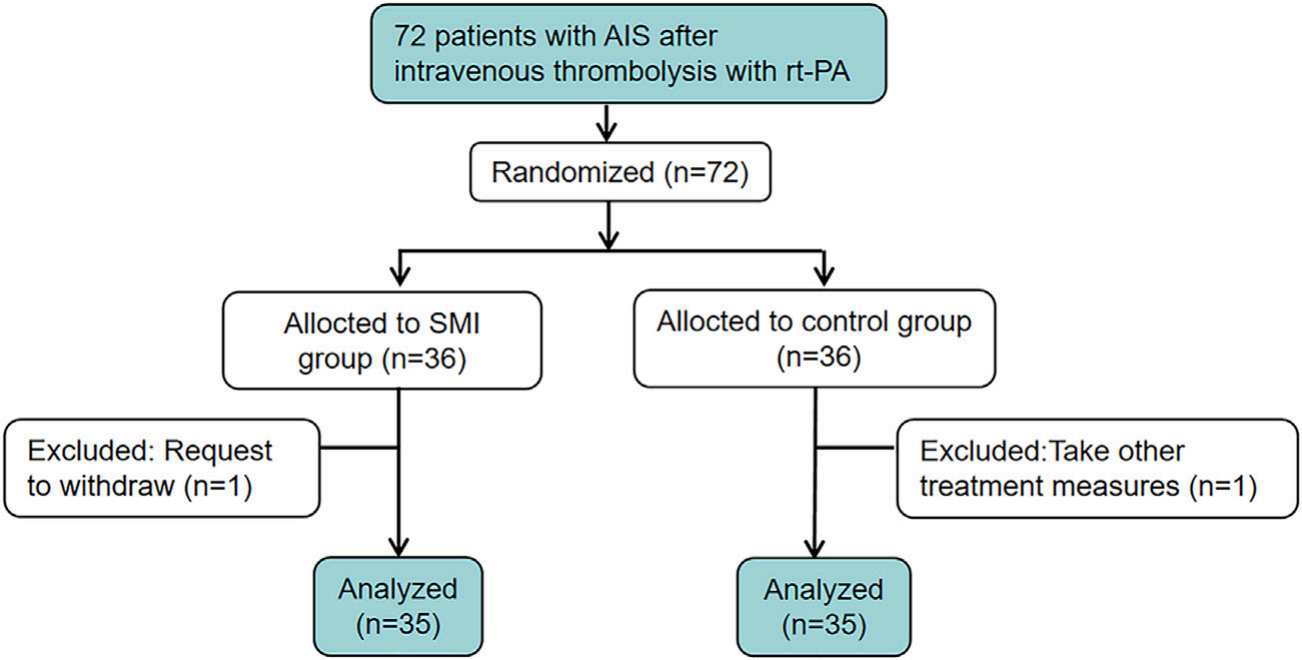
The clinical trial flowchart.

A total of 72 patients were enrolled in the study, with 36 participants assigned to the SMI group and 36 to the placebo group. During the trial, one participant withdrew from each group. (Figure 1). The baseline characteristics of the participants are summarized in Table 1. There was no significant difference in demographic and clinical characteristics between the two groups at baseline, indicating a well-balanced distribution.

**TABLE 1:** Baseline characteristics of the participants.

Clinical values	Placebo group (*n* = 35)	SMI group (*n* = 35)	*P*
Age (year)	64.71 ± 9.84	67.91 ± 11.99	0.226
Gender (Male)	19 (54.3%)	17 (48.6%)	0.632
Systolic pressure (mmHg)	147.83 ± 14.71	146.89 ± 18.43	0.775
Diastolic pressure (mmHg)	86.06 ± 8.14	82.69 ± 9.93	0.125
NIHSS (quartiles)	8 (7,9)	8 (7,9)	0.766
mRS (quartiles)	3 (3,4)	3 (3,4)	0.680
Anterior circulation infarction	30 (85.7%)	29 (82.9%)	0.743
Past medical history			
Stroke	9 (25.7%)	10 (28.6%)	0.788
Hypertension	28 (80.0%)	21 (60.0%)	0.068
Hyperlipidemia	8 (22.9%)	3 (8.6%)	0.101
Diabetes	8 (22.9%)	14 (40.0%)	0.122
Atrial fibrillation	3 (8.6%)	2 (5.7%)	0.643
Living habit			
Smoker	12 (34.3%)	9 (25.7%)	0.434
Alcohol	8 (22.9%)	6 (17.1%)	0.550

### 3.2 SMI improved the 30-day outcome in patients with IS receiving intravenous rt-PA thrombolysis

**FIGURE 2:**
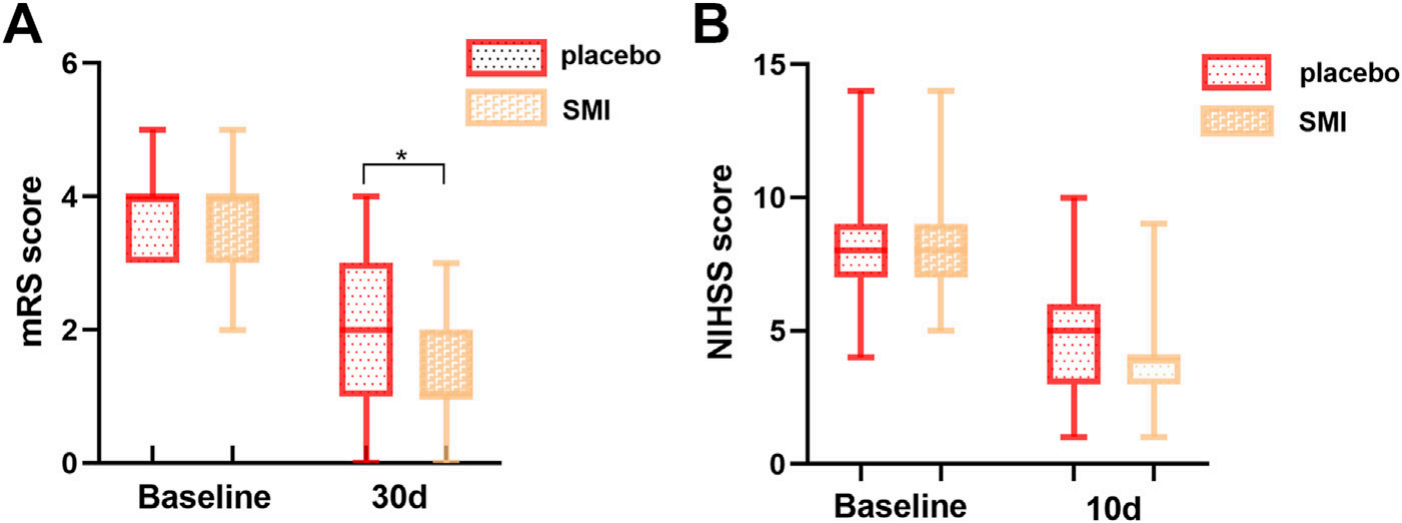
Comparison of scale scores of two groups of patients. **(A)** modified Rankin Scale (mRS) score. **(B)** National Institutes of Health Stroke Scale (NIHSS) score.

The primary outcome measure, the mRS Scale at 30 days post-onset, was significantly lower in the SMI group compared to the placebo group, indicating improved functional outcomes. However, no significant difference was observed between the two groups for the secondary outcome, the NIHSS score, assessed at day 10 post-treatment (Figure 2).

### 3.3 Safety of ten days of SMI injection

Serum biochemical indices, including liver and kidney function, were compared before and after treatment. The results indicated no statistically significant differences between the pre- and post-treatment values in either group (Tables 2, 3), suggesting that a 10-day course of SMI injection is safe.

**TABLE 2:** Baseline characteristics of serum biochemical indices of participants.

Name	Placebo group	SMI group	*P*
Triglyceride	1.33 (0.89,1.90)	1.31 (1.09,1.71)	0.92
Total Cholesterol	3.94 (3.52,4.77)	4.18 (3.58,5.05)	0.449
LDL-cholesterol	2.65 ± 0.92	2.61 ± 0.66	0.851
HDL-cholesterol	1.20 ± 0.33	1.29 ± 0.35	0.25
Homocysteine	12.1 (10.2,14.8)	12.5 (10.7,15.3)	0.626
uric acid	281.25 ± 82.39	281.06 ± 98.09	0.993
urea	5.26 (4.31,6.56)	5.8 (4.71,7.20)	0.184
AST	14.4 (10.9,27.5)	20.0 (16.0,33.0)	0.053
ALT	20.0 (14.1,23.0)	22.0 (16.0,27.0)	0.109
TBIL	14.62 ± 5.06	14.30 ± 5.71	0.802
DBIL	5.33 (3.90,6.55)	5.10 (4.50,7.21)	0.685
Scr	66.8 (54.0,79.5)	62.0 (53.0,78.4)	0.431

**TABLE 3:** Serum biochemical indices of participants after 10-day treatmen**t**.

Name	Placebo group	SMI group	P
Triglyceride	1.17 (0.97,1.70)	1.26 (0.86,1.46)	0.394
Total Cholesterol	3.46 (2.85,3.86)	3.82 (3.01,4.39)	0.069
LDL-cholesterol	2.02 ± 0.69	2.21 ± 0.53	0.189
HDL-cholesterol	1.11 ± 0.24	1.17 ± 0.23	0.309
Homocysteine	11.1 (8.4,12.5)	11.2 (8.8,13.6)	0.449
uric acid	255.77 ± 81.02	242.68 ± 74.18	0.483
urea	4.55 (3.56,5.24)	4.65 (3.54,5.26)	0.916
AST	24.0 (14.1,30.4)	25.2 (18.2,32.1)	0.428
ALT	21.4 (15.4,28.7)	23.0 (19.5,26.2)	0.411
TBIL	11.72 ± 4.13	13.86 ± 5.16	0.06
DBIL	4.39 (3.49,6.18)	5.20 (4.50,6.41)	0.113
Scr	65.8 (53.9,79.3)	62.4 (52.1,73.3)	0.363

### 3.4 Adverse events

Within 10 days of treatment, one patient in the placebo group experienced hemorrhagic transformation, two patients had gingival bleeding, and one patient experienced gastrointestinal bleeding. In the SMI group, two patients had hemorrhagic transformation, and two patients experienced a progressive stroke with deterioration following thrombolysis. The incidence of adverse events was not significantly different between the two groups.

### 3.5 SMI protects the cell viability of H2O2-stimulated PC12 cells

**FIGURE 3:**
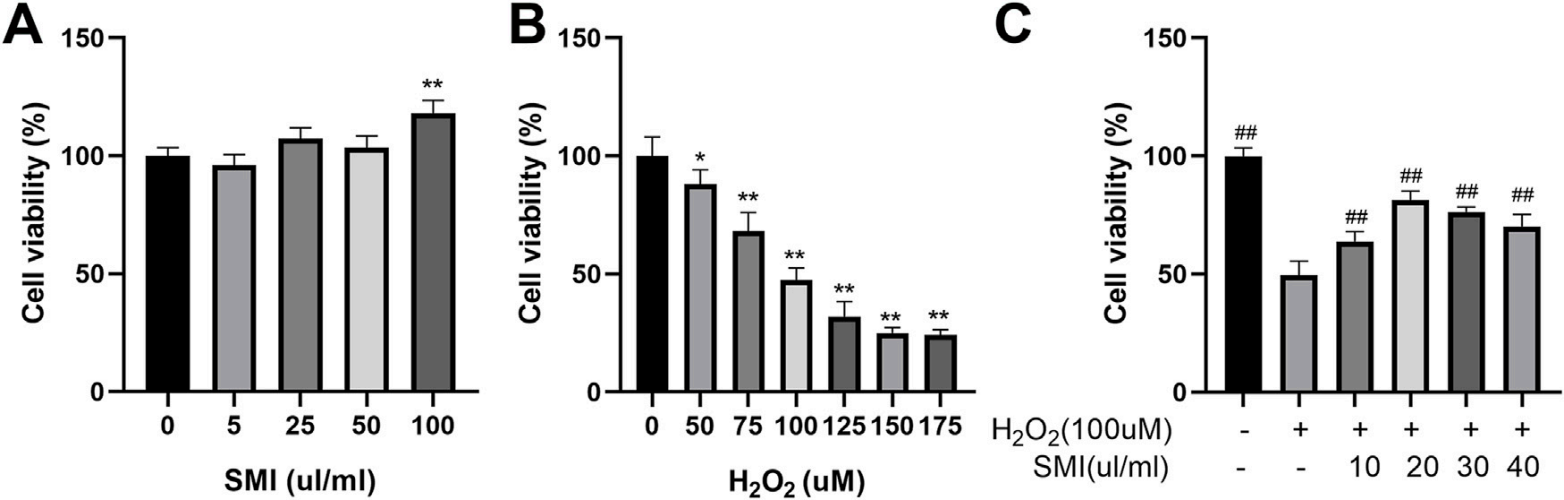
Shenmai Injection treatment enhances the viability of H2O2-induced PC12 cells. **(A,B)** Effects of different concentrations of H2O2 and Shenmai Injection on the viability of PC12 cells. **(C)** Effects of Shenmai Injection at different concentrations on the viability of PC12 cells exposed 10 µM H2O2. *P < 0.05 compared with the control group; **P < 0.01 compared with the control group; ##P < 0.01 compared with the H2O2-treated group. “+”: indicates the presence of treatments in cells; “−”: indicates the absence of treatments in cells.

The CCK-8 assay was used to evaluate the effects of varying concentrations of H_2_O_2_ and SMI on PC12 cell viability. As shown in Figures 3A,B), increasing concentrations of H_2_O_2_ led to a progressive decrease in cell survival rates. Conversely, SMI at concentrations ranging from 0 to 100 μL/mL did not significantly affect cell viability under normal conditions. When cells were exposed to 100 µM H_2_O_2_, the survival rate decreased to 45.56%. Based on these results, 100 µM H_2_O_2_ was selected to induce oxidative stress as a pathological model for subsequent experiments.

After inducing oxidative stress with 100 µM H_2_O_2_, cells were treated with different concentrations of SMI. Among the concentrations tested, 20 μL/mL SMI resulted in the highest cell survival rate (Figure 3C). Therefore, 20 μL/mL SMI was used in subsequent experiments.

### 3.6 SMI ameliorated cells injury by enhancing AMPKα1 level

**FIGURE 4:**
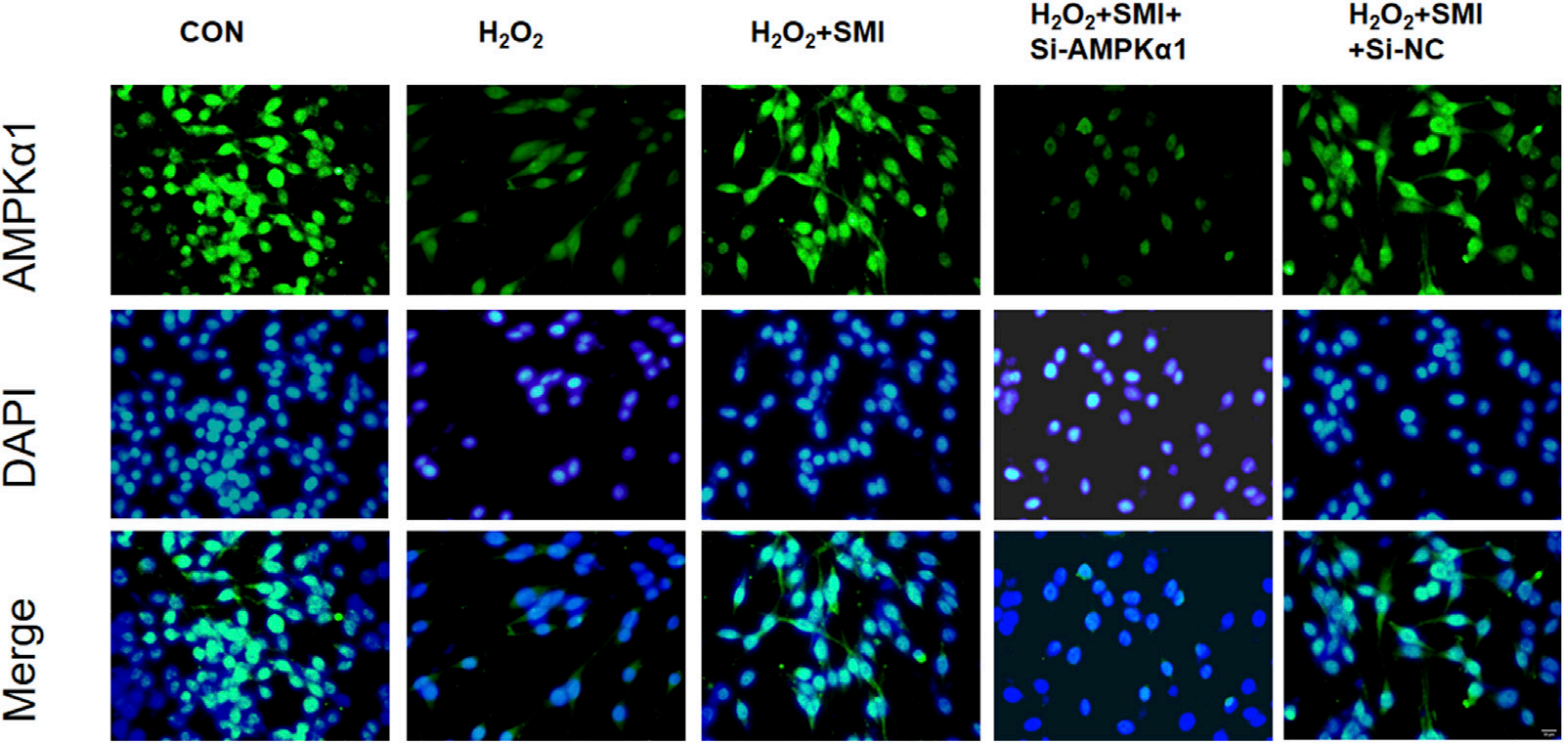
Immunofluorescence images showed the effect of SMI on AMPKα1 expression. Scale bar = 20 μM.

To clarify the mechanism of SMI, the expression of AMPKα1 was measured by immunofluorescence method, and to further investigate whether AMPKα1 plays the key role in SMI treatment, we used AMPKα1-specific siRNA to significantly decrease AMPKα1. As shown in Figure 4, SMI significantly enhanced the decreased fluorescence intensity due to H2O2 stimulation, and this effect was reversed by Si-AMPKα1. SiRNA-NC had no obvious effect on cells.

### 3.7 SMI attenuates H_2_O_2_-Induced Apoptosis in PC12 cells via AMPKα1

**FIGURE 5:**
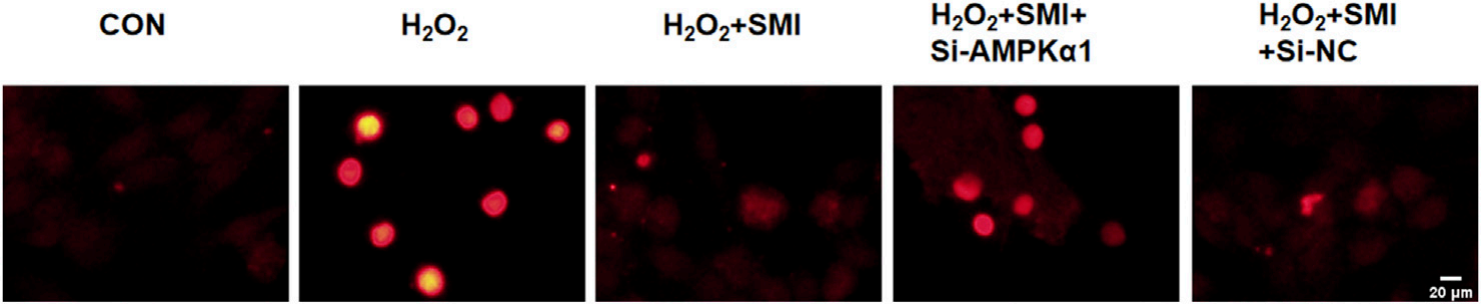
Shenmai Injection Attenuates H2O2-Induced Apoptosis in PC12 Cells via AMPKα1. Apoptosis detection using TUNEL assay, Scale bar = 20 μm.

The TUNEL assay was employed to detect apoptosis in PC12 cells. As shown in Figure 5, exposure to H2O2 significantly increased the proportion of TUNEL-positive cells (red fluorescence) compared to the control group. Treatment with SMI markedly reduced the apoptotic index relative to the H2O2 group.

Furthermore, the anti-apoptotic effect of SMI was negated by AMPKα1-specific siRNA (si-AMPKα1), indicating that AMPKα1 plays a critical role in the anti-apoptotic mechanism of SMI.

### 3.8 SMI attenuated H_2_O_2_-Induced Oxidative Stress in PC12 cells via AMPKα1

**FIGURE 6:**
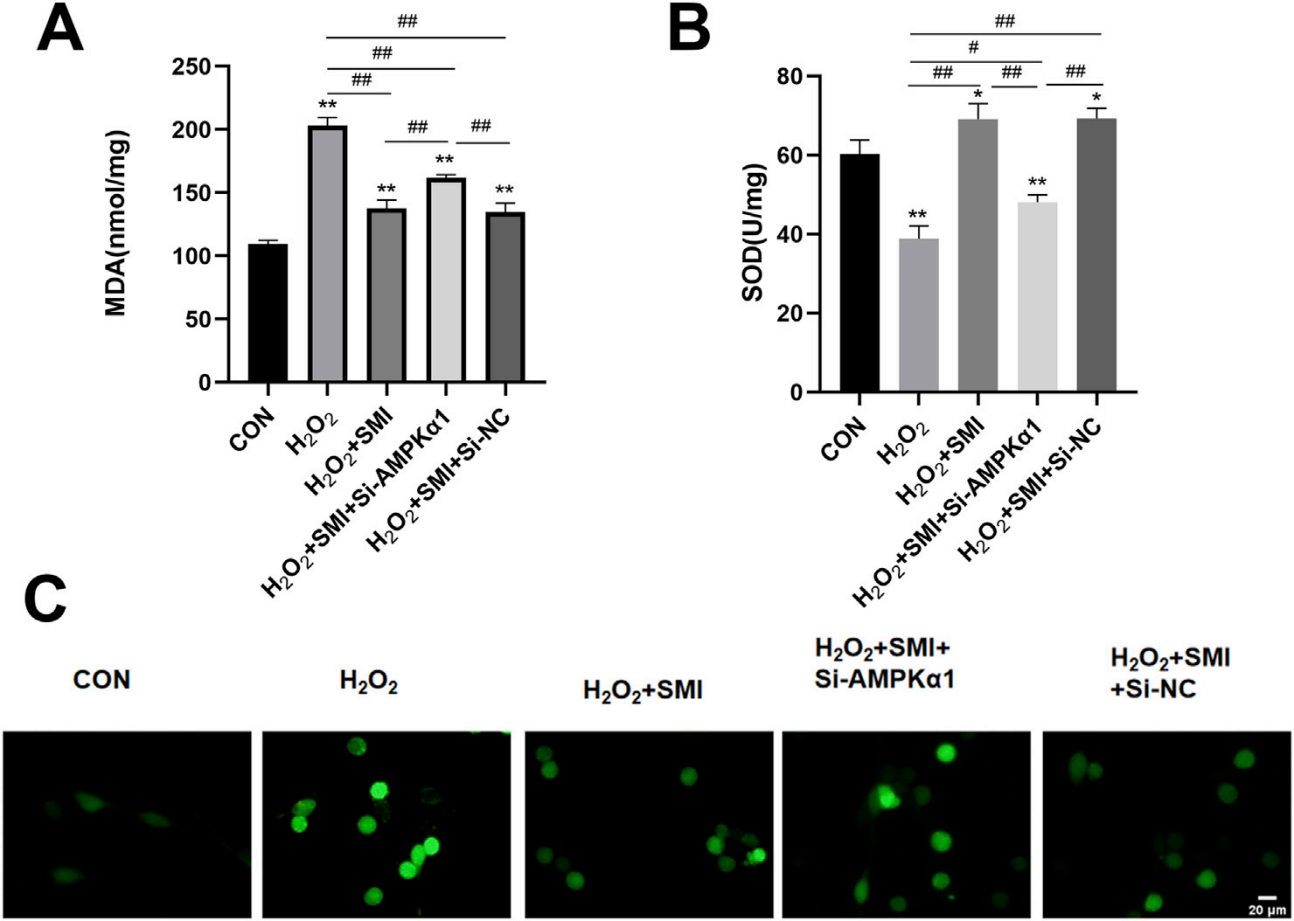
Shenmai Injection Attenuated H2O2-Induced Oxidative Stress in PC12 Cells via AMPKα1. **(A,B)** Levels of MDA and SOD in different group of PC12 cells. **(C)** Fluorescent image of ROS in different group of PC12 cells. Scale bar = 20 μm *P < 0.05 compared with the control group; **P < 0.01 compared with the control group; #P < 0.05; ##P < 0.01.

To investigate the potential mechanism of SMI’s protective effects against H_2_O_2_-induced injury, key markers of oxidative stress, including MDA, ROS, and SOD activity, were measured. Compared to the control group, H_2_O_2_ exposure significantly increased MDA and ROS levels while reducing SOD activity (Figure 6).

SMI treatment markedly increased SOD activity and decreased MDA and ROS levels in H_2_O_2_-stimulated cells. However, these antioxidative effects were inhibited by AMPKα1-specific siRNA (si-AMPKα1), highlighting the essential role of AMPKα1 in mediating SMI’s antioxidative properties.

### 3.9 SMI attenuated H_2_O_2_-Induced mitochondrial damage in PC12 cells via AMPKα1

**FIGURE 7:**
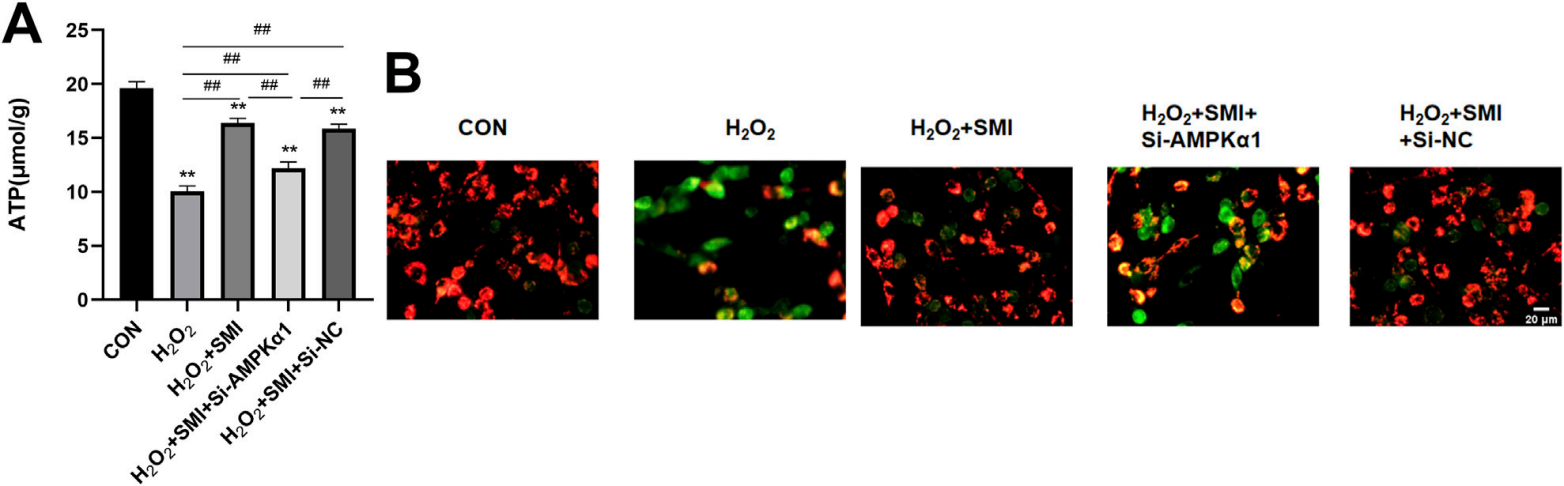
Shenmai Injection attenuates H2O2-induced mitochondrial damage in PC12 cells via AMPKα1. **(A)** Fluorescent images of mitochondrial membrane potential using JC-1 staining. Scale bar = 20 μm. **(B)** ATP levels in PC12 cells. **P < 0.01 compared with the control group; ##P < 0.01.

Mitochondrial function was assessed by measuring mitochondrial membrane potential using JC-1 staining and ATP levels. SMI treatment significantly shifted JC-1 fluorescence from green to red, indicating improved mitochondrial membrane potential, and increased ATP levels compared to the H2O2 group (Figure 7). However, these effects were partially reversed by si-AMPKα1, highlighting the role of AMPKα1 in the protective mechanism of SMI.

## 4 Discussion

In this study, we found that SMI could significantly reduce the mRS score after 30 days, indicating improved functional outcomes for patients with ischemic stroke. But no significant difference was found in the NIHSS score between the patients treated with SMI for 10 days and those in the placebo group. Additionally, there was no statistical differences in adverse events or safety indicators between the two groups, suggesting that SMI is safe for use in this context.

SMI treatment obviously improved cell viability. Further experiments confirmed that SMI exerted antioxidation effect by regulating MDA, SOD, and ROS levels or activities. These effects were accompanied by improvements in mitochondrial membrane potential and ATP content, ultimately preventing cell apoptosis, as evidenced by a decreased TUNEL-positive rate.

SMI is widely used in clinical treatment of coronary heart disease (Zhou and Jia, 2022), heart failure (Jia et al., 2022), myocardial infarction (Chen, 2019), cerebral infarction (Zhang et al., 2021). Numerous studies have shown that SMI can improve cellular tolerance to ischemia and hypoxia and enhances microcirculation (Tian, 2010; Zhuang et al., 2005). These findings align with our results, where SMI treatment improved the 30-day functional outcomes of IS patients. SMI is a Chinese patent medicine injection that has demonstrated significant neuroprotective effects in previous studies. Specifically, SMI and its main active components, ginsenosides Rb1 and Rg1, have been shown to reduce lactate dehydrogenase release and improve the survival of cultured neurons, vascular endothelial cells, and astrocytes under hypoxic/hypoglycemic/reoxygenation injury. (Zhang et al., 2005). Additionally, SMI helps maintain blood-brain barrier integrity during focal cerebral ischemic injury (Chen et al., 2018) and protects mitochondria from oxidative stress by increasing the level of pyruvate dehydrogenase (Zhao et al., 2018). These findings support the therapeutic potential of SMI in ischemic stroke and other ischemic conditions.

The AMP-activated protein kinase (AMPK), known as an “energy sensor” or “gauge,” is expressed across all types of cells and plays a crucial role in regulating cellular energy homeostasis. Previous studies have demonstrated that AMPK has a protective effect on global cerebral ischemia (Ashabi et al., 2014; Duan et al., 2019). AMPK is a heterotrimeric complex composed of α, β, and γ subunits, which respectively mediate enzymatic, scaffolding, and regulatory functions (Hardie, 2015; (Crozet et al., 2014). AMPKα1, an important subtype of AMPK, plays a pivotal role in arteriogenesis and collateral remodeling. Its activation has been shown to contribute to the recovery of occlusive vascular diseases (Zhu et al., 2016), and promotes the activity of antioxidant enzymes, such as SOD, which helps protect neurons from oxidative stress-induced damage and neuronal death (St-Pierre et al., 2006).

In this study, we observed that SMI could activate the AMPKα1 in PC12 cells exposed to oxidative stress. Furthermore, the protective effect of SMI on cell viability and apoptosis was partially inhibited following transfection with si-AMPKα1, supporting the notion that AMPKα1 plays a crucial role in mediating the neuroprotective effects of SMI. Our findings suggest that SMI may attenuate oxidative stress and apoptosis induced by H2O2 through the AMPKα1, highlighting a potential mechanism for its therapeutic effects in ischemic stroke.

However, our study has several limitations. First, the 10-day treatment period with SMI and the outcome evaluation conducted on the 10th day may not have been long enough to see significant differences in clinical outcomes. Previous studies suggest that a longer treatment duration, typically at least 14 days (Zhen, 2019), is required to observe more pronounced effects. Also, the relatively small sample size in our study could impact the statistical power of the results. A larger cohort in future studies would help to validate these findings. Another limitation is that our study only employed *in vitro* experiments to explore the potential mechanisms of SMI for IS, which provides a lower level of evidence. To better understand the pharmacological effects of SMI and confirm its clinical relevance, further *in vivo* studies and more comprehensive laboratory research are necessary. These additional studies should aim to elucidate the full scope of SMI’s mechanisms and its therapeutic potential in ischemic stroke. What’s more, according to traditional Chinese medicine theory, SMI is suitable for patients with Qi deficiency, but not for patients without Qi deficiency symptoms.

## Data Availability

The raw data supporting the conclusions of this article will be made available by the authors, without undue reservation.
