# Supplementary material for: Shenmai Injection enhances short-term outcomes in ischemic stroke patients after thrombolysis via AMPKα1
Source: Front Pharmacol. 2025 May 1;16:1552493. doi: 10.3389/fphar.2025.1552493 (PMC12078230; doi:10.3389/fphar.2025.1552493)
Supplement: Supplementary file 1 [file DataSheet1.docx]

Supplementary Material

# Supplementary Figures


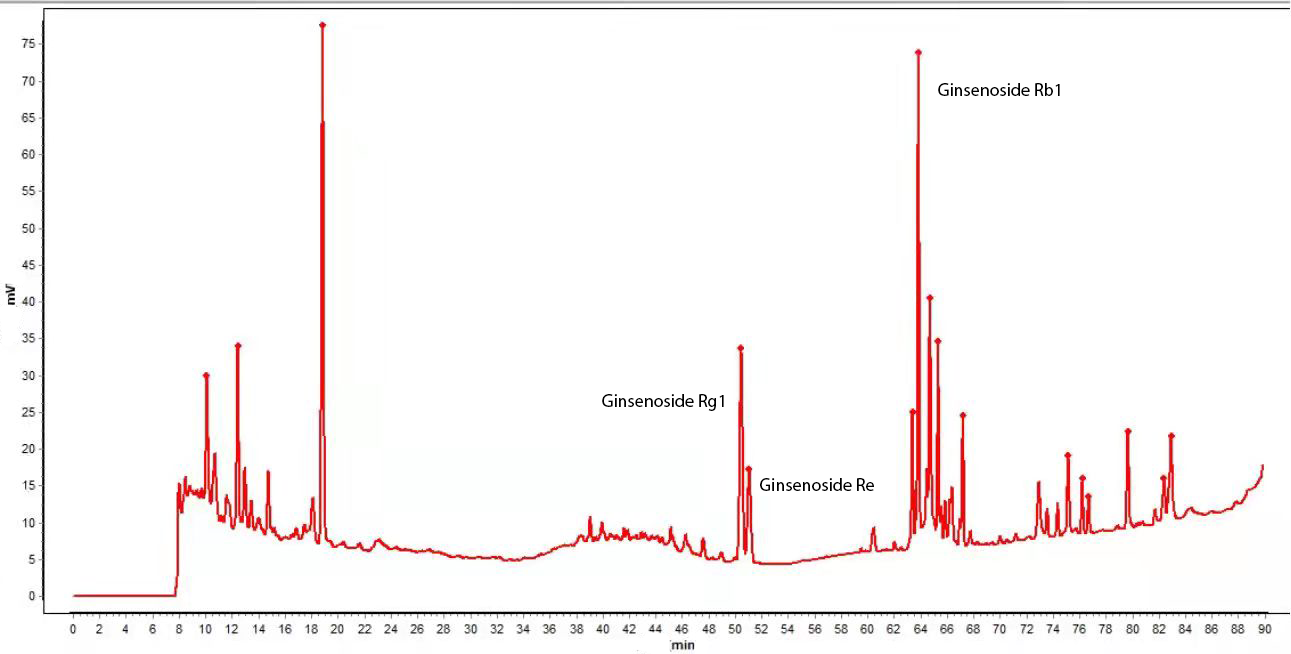


**Supplementary Figure S1.** **HPLC analysis of Shenmai Injection**. The quality control of the fingerprint of Shenmai Injection requires that 16 characteristic peaks corresponding to the standard fingerprint of Shenmai injection should be presented within 8-90 min. And clarify the three characteristic peak components, namely Ginsenoside Rb1, Ginsenoside Rg1 and Ginsenoside Re.
